# Sleep and Sedative States Induced by Targeting the Histamine and Noradrenergic Systems

**DOI:** 10.3389/fncir.2018.00004

**Published:** 2018-01-26

**Authors:** Xiao Yu, Nicholas P. Franks, William Wisden

**Affiliations:** ^1^Department of Life Sciences, Imperial College London, London, United Kingdom; ^2^Centre for Neurotechnology, Imperial College London, London, United Kingdom; ^3^UK Dementia Research Institute, Imperial College London, London, United Kingdom

**Keywords:** sedation, NREM sleep, zolpidem, GABA_A_ receptor, histamine, α2 adrenergic agonists, dexmedetomidine, xylazine

## Abstract

Sedatives target just a handful of receptors and ion channels. But we have no satisfying explanation for how activating these receptors produces sedation. In particular, do sedatives act at restricted brain locations and circuitries or more widely? Two prominent sedative drugs in clinical use are zolpidem, a GABA_A_ receptor positive allosteric modulator, and dexmedetomidine (DEX), a selective α2 adrenergic receptor agonist. By targeting hypothalamic neuromodulatory systems both drugs induce a sleep-like state, but in different ways: zolpidem primarily reduces the latency to NREM sleep, and is a controlled substance taken by many people to help them sleep; DEX produces prominent slow wave activity in the electroencephalogram (EEG) resembling stage 2 NREM sleep, but with complications of hypothermia and lowered blood pressure—it is used for long term sedation in hospital intensive care units—under DEX-induced sedation patients are arousable and responsive, and this drug reduces the risk of delirium. DEX, and another α2 adrenergic agonist xylazine, are also widely used in veterinary clinics to sedate animals. Here we review how these two different classes of sedatives, zolpidem and dexmedetomideine, can selectively interact with some nodal points of the circuitry that promote wakefulness allowing the transition to NREM sleep. Zolpidem enhances GABAergic transmission onto histamine neurons in the hypothalamic tuberomammillary nucleus (TMN) to hasten the transition to NREM sleep, and DEX interacts with neurons in the preoptic hypothalamic area that induce sleep and body cooling. This knowledge may aid the design of more precise acting sedatives, and at the same time, reveal more about the natural sleep-wake circuitry.

## Introduction

Every year, 250 million patients worldwide are given anesthetics; millions take sleeping medications to sedate themselves, and every day, at some point in the 24-h cycle, all humans and animals require natural sleep. Anesthesia, sedation and sleep are commonplace states that involve reversible loss of consciousness, but we do not understand how these processes work at a circuit level. Research suggests that at least sedation and deep sleep are connected mechanistically. Understanding how they interlink is important for both neuroscience and medicine. Sedatives target just a handful of receptors and ion channels (Franks, 2008; Steinberg et al., 2015; Wisden et al., 2017). But we have no satisfying explanation for how activating these receptors produces sedation. In particular, do sedatives act at restricted brain locations and circuitries or more widely (Franks, 2008; Rihel and Schier, 2013)?

In this review article, we describe how two specific drug systems induce sleep: positive-allosteric modulators of GABA_A_ receptors acting on histamine neurons in the posterior hypothalamus induce a natural NREM-like sleep; whereas α2 adrenergic agonists acting in the preoptic area (POA) of the hypothalamus induce a deeper form of NREM-like sleep, akin to recovery sleep following sleep deprivation. From studying these systems, we suggest that more can be learnt about the natural sleep-wake circuitry and potentially, better sedatives can be developed.

## Neuromodulation and the Waking State

The waking state is maintained by continual activation of aminergic (histamine, dopamine, noradrenaline (NA), acetylcholine), hypocretin/orexinergic (peptidergic), CART (peptidergic), and selective glutamatergic and GABAergic pathways (Wada et al., 1991; Carlsson, 2001; Gu, 2002; Berridge and Waterhouse, 2003; Anaclet et al., 2009; Lee and Dan, 2012; Sara and Bouret, 2012; Weber and Dan, 2016; Eban-Rothschild and de Lecea, 2017; Gradinaru, 2017; Jones, 2017; Lőrincz and Adamantidis, 2017; Lovett-Barron et al., 2017; Scammell et al., 2017; Schöne and Burdakov, 2017). When the brain is permeated with these neuromodulators we are wakeful and conscious (Gu, 2002; Constantinople and Bruno, 2011; Harris and Thiele, 2011; Arnsten et al., 2012; Lee and Dan, 2012; Krishnan et al., 2016; Lovett-Barron et al., 2017; van Kempen et al., 2017). In wakefulness, the cortical electroencephalogram (EEG) is desynchronized, fast and random, reflecting the variety of firing rates of hippocampal and cortical neurons (Harris and Thiele, 2011); neurons in the neocortex are persistently depolarized (Constantinople and Bruno, 2011), and there are no extended periods of synaptic silence (Constantinople and Bruno, 2011).

Wakefulness is a matrix of modalities (Harris and Thiele, 2011; Arnsten et al., 2012). It is difficult to say precisely what wakefulness is, except to note that without the action of neuromodulators, and if instead there was only glutamate and GABA in the hard-wired circuitry, we would be diminished in thoughts and actions, or even frozen (Carlsson, 2001; Gu, 2002; Robbins and Arnsten, 2009; Arnsten et al., 2012; Schöne and Burdakov, 2017). These neuromodulator actions are embedded in most aspects of circuitry function (Ellender et al., 2011; Arnsten et al., 2012). Neuromodulatory transmitters are often released extrasynaptically, and diffuse to extrasynaptic receptors, influencing many neurons simultaneously, a process termed volume transmission (Fuxe et al., 2010). A classic example is dopamine, which when lacking in Parkinson’s disease, results in severe impairments in the planning of movements and movement initiation, as well as cognitive dysfunctions. Dopamine is necessary to promote wakefulness (Cho et al., 2017; Eban-Rothschild and de Lecea, 2017; Oishi and Lazarus, 2017). Matsuda et al. (2009) illustrate the arborization of a single dopamine neuron in the substantia nigra—this axonal arbor fills a substantial volume of the caudate-putamen, and thus could influence many target cells and their associated circuitry (Matsuda et al., 2009).

## The Regulation of Natural Sleep

Sleep and wake are determined with polysomnography, which is to say by combining EEG and electromyogram (EMG) scoring, and in the case of larger mammals also electrooculographic (EOG) scoring (Pace-Schott, 2009). A mouse typically abruptly moves from wakefulness into a few minutes of NREM sleep, then a minute or so of REM sleep, then back to NREM sleep, then REM sleep, then wakefulness (Vyazovskiy and Harris, 2013). All mammals have these cycles, but the timings differ. In NREM sleep of rodents, the EEG is predominated by 0.5–4.5 Hz rhythms termed δ waves. In NREM sleep, muscle tone, heart rate, blood pressure and respiration rates are lower than during waking. NREM sleep transitions to REM sleep. In REM sleep, the EEG resembles that of the waking state, but skeletal (postural) muscle tone is at its lowest level—it is muscle atonia—resulting from inhibition of spinal motor neurons. During REM sleep, heart rate, blood pressure and respiration rates increase from their low NREM levels, and sympathetic nerve activity increases (Pace-Schott, 2009). Of the sleeping states, the NREM sleep period seems fundamental because it happens first (wakefulness to NREM) and lasts the longest. Most mammals demonstrate polyphasic sleep patterns distributed across their entire sleep-wake cycle, while humans are mostly mono/biphasic sleepers and concentrate most of their sleep time at night.

**Figure 1:**
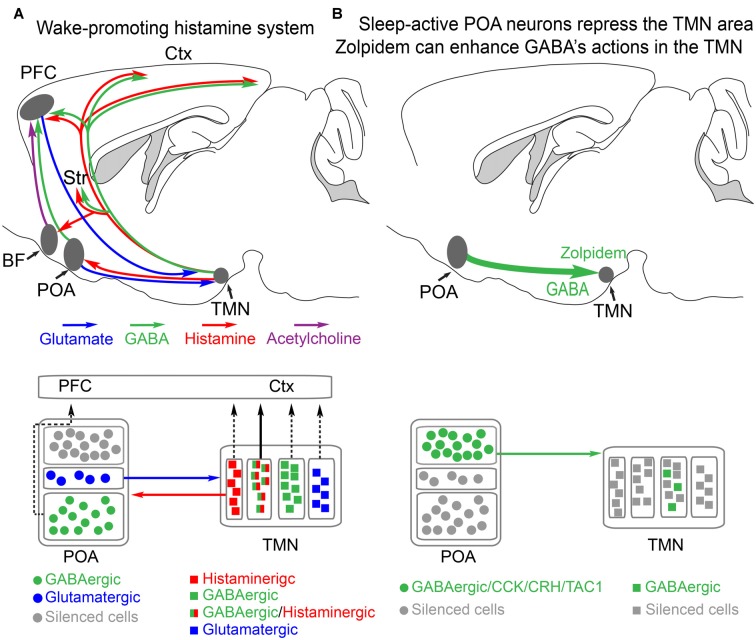
Histamine-GABA neurons in the tuberomammillary nucleus (TMN) area project widely to produce wakefulness and are silenced during NREM sleep by preoptic GABAergic neurons or by zolpidem. **(A)** Simplified view of how histamine/GABA neurons in the tuberomamillary area (TMN) of the posterior hypothalamus promote wakefulness. During the wake state, histamine/GABA neurons are active and their ascending histamine/GABA fibers release histamine (red) and GABA (green) into the prefrontal cortex (PFC), neocortex (Ctx) and striatum (Str, caudate-putamen). Glutamatergic pyramidal neurons in the PFC send excitatory projections to the histamine neurons in the TMN, reinforcing wakefulness. Histamine-only projections from the TMN also excite cholinergic neurons in the basal forebrain, and the axons of these excited cholinergic neurons release acetylcholine throughout the cortex. Histamine-only projections also excite GABAergic neurons in the preoptic area (POA) of the hypothalamus; these GABA neurons in turn are believed to supress sleep-active GABA neurons that project back to the TMN area that inhibit the histamine neurons. Within the POA, certain GABAergic cells project to the PFC and support wakefulness, and some glutamate cells in the POA also send excitatory projections to the histamine neurons to reinforce wakefulness. The TMN area also contains probably many wake-active glutamatergic and GABAergic neurons in addition to those that are histaminergic/GABAergic or histamine only. The contribution of these cells to wakefulness is not known. Based on Yu et al. (2015) and Chung et al. (2017). **(B)** During NREM sleep, the histaminergic neurons and probably some of the other cell types in the TMN area are inhibited by GABAergic projections (green) from the POA area. The GABAergic sleep active neurons probably co-release peptides, including CCK, CRH, TAC1 and galanin. The function of these peptides is not known. Zolpidem can also potentiate these GABAergic inputs via its actions at the postsynaptic GABA_A_ receptors on histamine neurons. Adapted from Uygun et al. (2016).

The firing of some aminergic and cholinergic neurons is vigilance state-dependent (Saper et al., 2010; Xu et al., 2015; Cho et al., 2017; Eban-Rothschild and de Lecea, 2017). These neurons are largely silent during NREM sleep, and sometimes active in REM sleep depending on the type of cell. NREM sleep emerges when neuromodulators are removed from the brain (Figure 1). Only a few years ago, there were just a few brain centers thought to actively promote NREM sleep. These included the ventral lateral preoptic area (VPLO), at the base of the hypothalamic POA (Sherin et al., 1996). The concept that preoptic GABAergic neurons inhibit wake-promoting aminergic neurons such as histamine neurons seems correct (Saper et al., 2010; Scammell et al., 2017), but much of the circuitry still needs to be elucidated (see “More on the Preoptic Area and the Induction of Natural NREM Sleep” section).

The number of neural sites that induce sleep when artificially stimulated has proliferated. Neural groups scattered through the neuroaxis, when selectively activated by chemo- or optogenetic methods, promote a transition from wakefulness to NREM sleep (Weber and Dan, 2016; Eban-Rothschild and de Lecea, 2017; Scammell et al., 2017). Particular interneuron subtypes in the mouse neocortex, when selectively activated, can also induce a NREM-like EEG (Funk et al., 2017), showing that sleep can be driven from top-down. How these circuits fit together, and if there is a “master NREM sleep-circuit” which drives the others is a challenge for the field to resolve. This task will not be straight-forward because of the extensive inter-connectivity of the brain, and multisite recordings of genetically specified cells will be needed to see if particular groups of cells lead others in the induction of NREM or REM sleep (see the study of Lovett-Barron et al., 2017 for an analogous approach to studying wake-promoting neurons). For example, we found using multisite recordings in the rat, that the central midline thalamic nuclei lead the neocortex in the appearance of delta waves in the transition from wakefulness to NREM sleep and propofol-induced anesthesia (Baker et al., 2014).

A critical point is that although stimulating a particular cell type might induce a vigilance state such as wake or NREM sleep, this does not necessarily mean that this cell type is physiologically involved in the induction or maintenance of this state. The dense interconnectivity of the brain can give misleading information on critical circuitry. This issue is well discussed in the article of Otchy et al. (2015) and by Sudhof (2015). It is important to evaluate the vigilance state-dependent activity of particular cell types. For example, if it is found that a particular cell type, when artificially activated induces NREM sleep, but mainly fires during wakefulness, this makes this cell type unlikely to be physiologically involved in triggering or maintaining sleep.

## More on the Preoptic Area and the Induction of Natural NREM Sleep

The POA in the hypothalamus has been the best studied for its sleep-wake promoting role (Sherin et al., 1996; Szymusiak et al., 2007; Takahashi et al., 2009). At the entry to NREM sleep various GABAergic neurons in the POA become more active (Takahashi et al., 2009). These neurons send GABAergic projections to the ascending aminergic systems, such as the histamine neurons in the tuberomammillary area in the posterior hypothalamus and orexin neurons in the lateral hypothalamus (Sherin et al., 1996; Chung et al., 2017; Figure 1A). When these GABAergic neurons are active, the arousal-promoting histamine and orexin neurons are supressed (Sherin et al., 1996; Saper et al., 2010; Scammell et al., 2017).

The VLPO area was defined as a sleep-promoting nucleus on the basis of c-FOS activation, but does not have any neurochemical markers to define it. In the rat, the expression of the neuropeptide galanin marks out GABAergic sleep-promoting cells. But in the mouse, galanin expression is widely distributed, and stimulating galanin cells in the mouse POA produces wakefulness rather than NREM sleep (Chung et al., 2017). Additionally, in the mouse a larger area of the preoptic hypothalamus than the VLPO area seems to be involved in NREM sleep regulation, as defined by activity-tagging with c-FOS expression (Zhang et al., 2015). We think that cells in the broader POA, rather than just those in VLPO, regulate sleep. The POA includes the medial and median preoptic neurons, which also induce NREM sleep (Zhang et al., 2015).

There is much we do not know about how the POA regulates sleep-wake behavior. For example, why the sleep-promoting POA GABAergic neurons become active in the first place, and stay active, and then switch off when sleep ends, remains unknown. There is no definitive view on precisely what the biochemical signal to sleep during sleep deprivation is—clearly this is fundamental, and there could be multiple signals which include adenosine accumulating extracellularly in the basal forebrain (Porkka-Heiskanen, 2013). The POA neurons are regulated by homeostatic, circadian and metabolic inputs (e.g., satiety promotes sleep; Kapás and Szentirmai, 2014; Eban-Rothschild and de Lecea, 2017), but what these inputs precisely are unknown. In mice, heterogeneous types of preoptic GABAergic neurons co-release peptides such as CCK and dynorphin to initiate NREM sleep (Chung et al., 2017). The function of the peptides is unknown. Other GABAergic neurons in an (undefined) POA region of the mice, including ones that co-release galanin, can promote wakefulness (Chung et al., 2017; Figure 1A). There remains considerable work to delineate the POA and how it integrates and outputs all of its homeostatic functions (e.g., sleep-wake, body temperature, electrolyte balance, aggression, sex). It is not understood how the brain moves from NREM to REM sleep and back again, or even why it wakes up.

## The Purpose of Sleep

The purpose of sleep remains a mystery, but everyone agrees, and indeed knows from their own experience, that sleep seems restorative (Walker, 2017). Indeed, the homeostatic sleep drive ensures that there is a restoration to partially catch up on lost sleep. The restoration hypotheses encompass cognitive memory and rebalancing synaptic strengths, motor performance, metabolite clearance, the immune system and in setting the level of metabolism or optimizing energy allocation to different organs and processes (Vyazovskiy and Harris, 2013; Xie et al., 2013; Schmidt, 2014; Schmid et al., 2015; Mander et al., 2016; Cirelli and Tononi, 2017; Walker, 2017). The timing of sleep onset and its end is, in part, believed to be determined by when homeostatic corrections are needed or have been completed, and partly on the circadian cycle (Borbély et al., 2016). In fact, the best way to induce deep sleep is by sleep deprivation. If sleep deprivation is extreme enough, deep sleep is unavoidable and this drive to sleep overrides any wake-promoting effect from the circadian system. Sleep deprivation is not a healthy or convenient way to comfortably induce sleep, but it is a physiological route whose mechanism might 1 day be mimicked by an, as yet, undeveloped drug once we know more about the circuitry involved. Meanwhile, there are several good drugs already available, which as we shall see in the next sections, selectively intervene with the wake-promoting neuromodulators histamine and NA to induce NREM sleep.

## Sleeping Medication and Sedatives

Sedative drugs are used both to treat insomnia (Nutt and Stahl, 2010; Greenblatt and Roth, 2012; Wisden et al., 2017), but also for investigative procedures and in long-term intensive care (Ramsay et al., 1974). For primary insomnia, the current clinical recommendation is to try cognitive behavioral therapy before trying drugs (Trauer et al., 2015; Kripke, 2016). The use of sleep-promoting drugs for treating primary insomnia has been criticized on the grounds that these drugs often do not work much better than placebos and that they undermine health (Kripke, 2016; Walker, 2017). There is a claim that hypnotics increase the risk of death by 3–6-fold in otherwise healthy people, possibly by increasing the susceptibility to infection (Kripke et al., 2012). This has recently been disputed (Patorno et al., 2017); a study that data-mined the clinical outcomes of over 1.2 million people in a de-identified commercial database found no evidence that benzodiazepines increased mortality (Patorno et al., 2017). The use of sedatives in the intensive care context is uncontroversial. Sedatives administered during intensive care help patient compliance, reduce patient distress and delirium, and promote sleep.

Operationally and experimentally, the sedated state comprises a reduced movement and eyes closed (for humans), an increase in slow (0.5–4.5 Hz) oscillatory δ power in the EEG, reduced breathing rate, and reduced body temperature, but still with functional respiratory and brainstem circuitry and slow cognitive responsiveness. It is usually possible to be aroused from sedation, just like from natural sleep. Chemically-induced NREM-like sleep (i.e., sedation) is not identical to natural NREM sleep. But various types of sedatives/sleeping medications do work at selective nodes of the sleep-wake circuitry to induce states partially resembling aspects of NREM sleep (see below).

Beyond sedation, there is surgical general anesthesia (Franks, 2008; Brown et al., 2010; Mashour and Hudetz, 2017). This is a state where the patient or animal is unconscious, immobile, and insensitive to pain. Brain stem and hypothalamic reflexes are inhibited and patients need active support of their cardiorespiratory and thermoregulatory systems (Brown et al., 2010) General anesthesia does not resemble sleep (Akeju and Brown, 2017). In these general anesthetic states, the EEG is either flat (isoelectric) or exhibits “burst suppression”—large amplitude fast spikes interspersed with a flat EEG (Brown et al., 2010). A good illustration of burst suppression recorded in the EEG under general anesthesia can be seen in Figure 6iii in the study of Reynolds et al. (2003), and in Figure 1 from Brown et al. (2010).

General anesthesia emerges from both top down (neocortical) and bottom up anesthetic-induced inhibition of many types of circuitry (Mashour and Hudetz, 2017). Under general anesthesia the subject cannot be aroused, except when the drug is wearing off (after clearance and metabolism) or by terminating its action with an antagonist. In laboratory rodents, stimulating the noradrenergic locus coeruleus (LC) or dopaminergic neurons in the ventral tegmental area (VTA) induces arousal from the anesthetized state (Vazey and Aston-Jones, 2014; Taylor et al., 2016). Thus, neuromodulators that promote wakefulness under natural conditions, if they are released at sufficiently high levels, such as provoked by chemogenetic or optogenetic stimulation, are powerful enough to overcome the general anesthetic effects of artificially strong GABAergic inhibition.

## Histamine and Wakefulness

Histamine stimulates aspects of wakefulness, such as motivation and cognition (Haas and Panula, 2003; Anaclet et al., 2009; Torrealba et al., 2012). Selective pharmacogenetic stimulation of histamine neurons promotes increased movement (Yu et al., 2015). Even before it was discovered that histamine was a transmitter in the brain, anti-histamines (i.e., H1 receptor antagonists) were noted to be sedatives (Monnier et al., 1967; Nicholson et al., 1991). H1 receptor antagonists, for example doxepin (a selective H1 antagonist at low concentrations), are making a comeback to treat primary insomnia (Yeung et al., 2015). Mice without the histamine synthesizing enzyme histidine decarboxylase (*hdc*) become easily drowsy in novel environments (Parmentier et al., 2002), although the effect is most pronounced at the diurnal light to dark transition, where *hdc* knockout mouse fail to show the usual increase in activity. The mismatch in phenotypes between acute pharmacology (H1 receptor antagonist) and chronic *hdc* gene knockouts, suggest compensations in the knockouts. In fact, selective optogenetic inhibition of histamine neurons produces an immediate transition to NREM sleep (Fujita et al., 2017).

Histamine neurons are located solely in a posterior hypothalamic area, the tuberomammillary nucleus (TMN), and send their axons throughout the brain (Panula et al., 1984; Köhler et al., 1985; Staines et al., 1987; Wada et al., 1991; Figure 1A). In the rat, there are about 2500 histamine neurons on each side of the brain (Köhler et al., 1985). Units in the TMN area, presumably the histamine neurons, seem selectively wake-active, and they start to fire, at around 5 Hz, just after wakefulness commences, so histamine neurons do not initiate wakefulness *per se* (Takahashi et al., 2006; Sakai et al., 2010). The vigilance-state dependence of histamine neurons has not been tested with genetically specified recordings, e.g., with GCaMP photometry selectively for histamine neurons, so it is possible that some of the wake-active neurons in the TMN area are not histamine neurons. There are other neuronal types in the TMN area (Figure 1B), glutamatergic and GABAergic neurons, and the vigilance state-dependent firing of these cells, or their precise identity has not been elucidated.

Wake-active hypocretin/orexin neurons provide a major excitatory drive onto histamine neurons (Eriksson et al., 2001; Schöne et al., 2014), and this could be a key way that orexin promotes arousal, amplifying its effects through the histamine system. On the other hand, in *hdc* knockout mice optogenetic stimulation of hypocretin/orexin neurons still promotes wakefulness from NREM sleep (Carter et al., 2009), so this wake-promoting route from orexin via histamine neurons has probably been compensated for in the long-term knock-out mice. Systemic administration of a dual orexin receptor antagonist, DORA-22, a hypnotic, acutely reduces histamine levels in multiple brain regions (prefrontal cortex (PFC), lateral hypothalamus), again emphasizing the difference in outcome between chronic genetic knockouts and acute pharmacological manipulations (Yao et al., 2017).

There are few histamine synapses (Takagi et al., 1986), and histamine’s main action is by volume transmission (Haas and Panula, 2003; Fuxe et al., 2010). Histamine is cleared from the extracellular space by reuptake into astrocytes by a transporter, the organic cation transporter 3, and then inactivated by methylation by histamine N-methyltransferase (Haas and Panula, 2003; Yoshikawa et al., 2013), which is found in the cytosol of astrocytes (Yoshikawa et al., 2013). As usual with modulatory actions, histamine excites neuronal networks in diverse ways: many small excitatory effects on different cell types and synaptic inputs sum into arousal-promoting effects (Bolam and Ellender, 2016).

Histamine activates excitatory metabotropic H1 and H2 receptors to trigger increases in Ca^2+^ and cAMP respectively (Panula et al., 2015). Effects of metabotropic histamine excitation include membrane depolarization and phosphorylation of voltage-gated K^+^ channels or decreasing the activity of K^+^ leak channels (Atzori et al., 2000; Ellender et al., 2011; Vu et al., 2015; Bolam and Ellender, 2016). H3 receptors, inhibitory metabotropic receptors that inhibit voltage-activated Ca^2+^ channels, are on the terminals of many types of neurons, as well as histaminergic axons themselves, which lead to reduced transmitter release of e.g., acetylcholine, GABA, glutamate and histamine (Panula et al., 2015).

There is likely to be feedback from the PFC down onto the ascending arousal systems. Prefrontal pyramidal neurons send glutamatergic projections to the histamine and other aminergic systems, where they could activate local inhibitory interneurons that then reduce the activity of the ascending aminergic cells (Wouterlood et al., 1987; Sara and Hervé-Minvielle, 1995; Riveros et al., 2015). There is also believed to be direct positive feedback from the PFC onto histamine neurons (Riveros et al., 2015). No circuitry details are available, and this needs to be elucidated with functional mapping.

## Local Circadian Clock Regulation of Histamine Production

**Figure 2:**
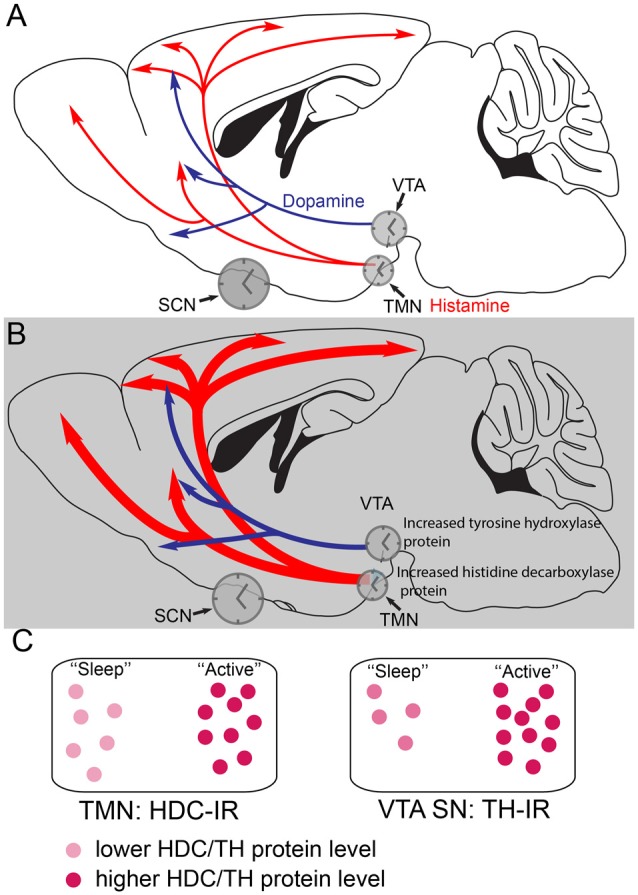
Histamine and dopamine synthesis are under local circadian control. **(A)** During the less active period (the “lights on” period) of mice, the dopaminergic and histaminergic cells have lower levels of transmitter synthesis for dopamine and histamine because the levels of tyrosine hydroxylase (TH) in the ventral tegmental area (VTA) and histidine decarboxylase (HDC) in the TMN are lower. The gene expression levels are controlled by clock genes in local circadian processes coordinated by the suprachiasmatic nucleus (SCN). **(B)** During the more active period of mice, the dark period, in histamine and dopamine cells levels of the TH and HDC proteins are higher, giving the potential for more transmitter release. **(C)** The relative levels of HDC and TH enzymes, as detected by immunoreactivity (IR) are shown schematically at the different times of day. Adapted from Chung et al. (2014) and Yu et al. (2014).

The histamine system is under local circadian control by the core circadian transcription factor BMAL1. The mRNA levels of the gene encoding the enzyme which makes histamine, HDC, varies with time of day (Yu et al., 2014), giving a clear difference in immunocytochemical staining for HDC depending on time of day, the levels of protein being highest during the “lights off” or dark period in the animal house (Yu et al., 2014; Figure 2A). In mice, the level of *hdc* mRNA anticipates this peaking of protein levels, with highest *hdc* transcription just before the dark period, and giving an increase in the apparent number of HDC immunopositive cells during the dark period (McGregor et al., 2017), probably because of the levels of HDC protein oscillating, and going below the detection limit for immunocytochemistry in some cells during the light period.

Selective deletion of the mouse *Bmal1* gene from the mouse histaminergic neurons abolishes the diurnal oscillation of *hdc* transcript levels, causing a sustained higher level of *hdc* transcription (Yu et al., 2014), so that HDC protein levels remain elevated, flattening out histamine levels in the brain. The result is that histamine levels are higher during the “rest period” of the mice so that they have severe sleep-wake fragmentation, with intrusions of wakefulness into sleep (Yu et al., 2014). These oscillations are probably coordinated by the master clock in the suprachiasmatic nucleus (SCN) in the anterior hypothalamus.

Dopamine production in the VTA is also regulated by a local circadian clock (Chung et al., 2014). Levels of the enzyme tyrosine hydroxylase (TH) in VTA dopamine neurons are under control of local clock transcription factors. TH protein levels are highest during the active period of the mice (Chung et al., 2014; Figures 2B,C). In fact, local circadian clocks are ubiquitous in the brain (Kyriacou and Hastings, 2010), probably serving to amplify the circadian signals from the SCN master clock, and ensuring that optimal levels of transmitters or circuit functions are available during particular behaviors, for example, in the case of histamine, ensuring that histamine-dependent neuromodulation is optimized during the period when animals are most active (Figure 2).

## Histamine-GABA Co-release

Neurons often co-release different transmitters (Tritsch et al., 2016; Ma et al., 2018). For example, excitatory orexin neurons release both glutamate and orexin onto the histamine neurons (Schöne et al., 2014; Schöne and Burdakov, 2017). In turn, some histamine neurons have the capacity to synthesize GABA (Vincent et al., 1983; Takeda et al., 1984; Köhler et al., 1985), and express the *vgat* gene (Yu et al., 2015), whose protein transports GABA into synaptic vesicles. Histamine is transported via the vesicular monoamine transporter (VMAT) protein into vesicles. These neurons co-release GABA with histamine. It is not clear if the transmitters are in the same vesicles or separate “histamine-only” and “GABA-only” vesicles. Optogenetic activation of genetically specified histaminergic axons in the neocortex triggers non-synaptic GABA release (which still occurs in the presence of histamine receptor antagonists and is thus not due to histamine activating GABAergic interneurons, which in turn would release GABA; Yu et al., 2015).

The GABA released from histamine axons activates predominantly extrasynaptic GABA_A_ receptors to produce a tonic inhibition, which is a form of GABA_A_ receptor inhibition that is not precisely time locked, and provides a form of gain control to the circuitry (Brickley and Mody, 2012; Yu et al., 2015). This tonic inhibition by GABA originating from histamine axons is, like the histamine signal, a form of volume transmission broadcast throughout the network. We proposed that this tonic inhibition might sharpen cognition/arousal by increasing the precision of action potential timing (Bright et al., 2007; Duguid et al., 2012; Wlodarczyk et al., 2013). The diffuse GABA originating from the histamine axons might also act as a break on wakefulness. Mice with *vgat* expression selectively knocked down in histamine neurons are considerably more active during the “lights-off”/dark period (Yu et al., 2015).

Not all histamine neurons release GABA, but their effect is passed on as inhibitory at the local network level because these “histamine only” axons excite GABAergic cells in the preoptic hypothalamus (Williams et al., 2014). Whether these GABA neurons are the interneuron GABA cells, which are in turn inhibiting sleep-active GABA cells (Liu et al., 2010), or are the ascending GABAergic cells from the preoptic hypothalamus which promote wakefulness (Chung et al., 2017), is unclear. A histaminergic projection from the TMN goes to the basal forebrain, a small nucleus in the vicinity of the POA (Figure 1A). In the basal forebrain, various types of neuron, GABA, acetylcholine and glutamate send axons to the neocortex to promote wakefulness (Xu et al., 2015; Jones, 2017; Yang et al., 2017). Lesioning the cholinergic basal forebrain neurons reduces histamine’s ability to promote wakefulness (Zant et al., 2012; Figure 1A). On these cholinergic neurons, histamine activates H1 receptors that then close leak TASK3 potassium channels, thus causing depolarization and activation of these cholinergic cells, and hence more wakefulness (an increase in high frequency EEG frequencies; Vu et al., 2015). Given that histamine fibers are found throughout the neocortex and hippocampus, there are probably parallel routes by which histamine promotes wakefulness, independent of the basal forebrain. To sum up, in this section, we have seen how effective the histamine system is in maintaining wakefulness. In the next sections, we will examine how selective inhibition of this “histamine hub” by drugs can promote sleep.

## GABA_A_ Receptor Positive Allosteric Modulators—Zolpidem, the Most Commonly Used Sleeping Medication

For primary insomniacs, those people that cannot get to sleep, and who do not succeed with cognitive behavioral therapy, the most popular (controlled) drug is an imidazopyridine compound, zolpidem (Ambien; Arbilla et al., 1985; Depoortere et al., 1986; Nicholson and Pascoe, 1986; Nutt and Stahl, 2010; Wisden et al., 2017). Previously, benzodiazepines, and before that barbiturates, were often the main drugs used to treat insomnia but they produced many off-target effects (Wisden et al., 2017). In contrast to most benzodiazepines, zolpidem has a rapid onset and short plasma half-life, giving it reduced “hangover effects” (Nutt and Stahl, 2010). There is, however, a risk of addiction (Janhsen et al., 2015). And especially for the elderly, there is a risk that taking zolpidem promotes falls and accidents if awakening occurs on the drug, although less so than for patients taking benzodiazepines (Allain et al., 2005).

Like benzodiazepines and barbiturates, zolpidem is a positive allosteric modulator at GABA_A_ receptors, which are GABA-gated chloride channels, but unlike benzodiazepines it works on a more limited range of GABA_A_ receptor subtypes, namely α1βγ2, α2βγ2 and α3βγ2-type GABA_A_ receptors, with a 20-fold preference in affinity for α1βγ2-type receptors (Pritchett and Seeburg, 1990). In practice, this means that zolpidem will probably work on all three GABA_A_ receptor subtypes *in vivo*. In the clinic, to counter the actions of any overdoses of benzodiazepines or zolpidem, their actions can be reversed by administering the benzodiazepine antagonist flumazenil (McCloy, 1995; Whitwam and Amrein, 1995). This drug binds to the same site between the α and γ2 subunits as the benzodiazepines and zolpidem (Wisden et al., 2009, 2017). Zolpidem does not gate the receptor alone, but requires GABA to be also bound at the receptor (Wisden et al., 2009). Zolpidem increases GABA’s efficacy at the receptor. Thus, for zolpidem to work, ongoing GABAergic transmission is required. The action of zolpidem is quite subtle in boosting this ongoing transmission. Zolpidem prolongs the duration of inhibitory postsynaptic currents by about 50% (Cope et al., 2004).

In humans, zolpidem’s net effect is to reduce the onset time (latency) to NREM sleep (Greenblatt and Roth, 2012). In animals and humans, zolpidem-induced NREM sleep is lighter than natural NREM. The power of the EEG in the frequency ranges 5–16 Hz is lower with zolpidem compared with natural NREM sleep, and in the β range (centered around 20 Hz) it is higher (Kopp et al., 2004; Uygun et al., 2016). Nevertheless, zolpidem induces synchronization in the 2–4 Hz (γ) range of frequencies. Pragmatically, it is unclear if these differences in the “zolpidem EEG” and the natural “NREM sleep” EEG are important. But there is a view that a perfect sedative would induce the natural NREM sleep state (Walker, 2017).

## Zolpidem and the Suppression of the Wake-Promoting Histamine System

The GABA_A_ receptors that are enhanced by zolpidem are expressed widely in the brain (Laurie et al., 1992; Wisden et al., 1992; Fritschy and Mohler, 1995). It might be thought that zolpidem acts all over the brain to induce NREM sleep, with IPSCs at many types of synapses throughout the brain summating to induce a non-specific state of sedation. However, this is not necessarily the case. A drug that can inhibit the hub-like arousal promoting neurons will be able to exert particular influence, because neurons such as histamine have a disproportionate effect on brain vigilance states (Wada et al., 1991). Surprisingly, chronically removing, by genetic ablation, the GABA_A_ receptors selectively from histamine neurons had no effect at all on the basal sleep wake cycle—the only phenotype was the mice took longer to go to sleep in a novel environment (Zecharia et al., 2012). As in other instances, the lack of effect on the sleep-wake cycle was probably another example of compensation resulting from chronic loss of a component. The system has time to adapt and implement other strategies. This plasticity is interesting and remarkable, but usually tends to be regarded as a confound in the interpretation of studies. In fact, injecting muscimol into the posterior hypothalamus of cats and rats induces NREM sleep/sedation (Lin et al., 1989; Nelson et al., 2003).

A customized pharmacogenetic approach enabled us to specifically examine the whole animal effects of reversibly increasing GABA_A_ receptor function, cell type selectively. By mutating a key amino acid (F77 changed to I77) in the GABA_A_ receptor γ2 subunit gene contributing to the zolpidem binding site, γ2F77I knock-in mice were made. In these mice, all of their GABA_A_ receptors are insensitive to zolpidem but still respond normally to GABA (Cope et al., 2004; Wulff et al., 2007). The zolpidem-sensitive γ2F77 subunit can then be selectively genetically swapped into selected neural cell types, such that the GABA inputs onto these cell types then become selectively zolpidem-sensitive (Wulff et al., 2007; Wisden et al., 2009). If this approach is done for histamine neurons in the TMN to make histamine neurons selectively zolpidem sensitive, and then zolpidem is given systemically to these mice, the latency to NREM sleep is strongly reduced (Uygun et al., 2016). Thus, a slight (50%) prolongation of the GABAergic IPSCs by zolpidem onto the histamine neurons, thus increasing the GABAergic drive onto these cells, is enough to increase the probability that the animals will enter NREM sleep and also double the amount of NREM sleep (Uygun et al., 2016). This fits with the original proposal that natural NREM sleep is in part induced by increased GABAergic drive onto histamine (and other aminergic neuronal types) to induce sleep (Sherin et al., 1996; Saper et al., 2010; Figure 1B). Of course, this does not mean that zolpidem actually works like this; probably various pathways are influenced by zolpidem, but at least in principle, zolpidem does not need to work all over the brain to trigger sedation. As discussed above, because of the dense inter-connectivity of the brain, triggering a chemogenetic response by inhibiting or activating a particular cell type does not necessarily imply that this cell type is involved in that behavior, or that a physiological process is being mimicked (Otchy et al., 2015).

Another feature of this zolpidem-induced NREM sleep by selectively increasing the inhibition of histamine neurons, is that its EEG power is the same as that of natural NREM sleep (Uygun et al., 2016). In contrast, the NREM sleep evoked by zolpidem when zolpidem can act on receptors throughout the brain has lower power than natural NREM sleep in the range above 5 Hz, and is higher in the 20 Hz range (Winsky-Sommerer, 2009; Uygun et al., 2016). This could mean zolpidem-induced sleep is less deep than natural sleep. So selectively inhibition of histamine neurons might allow a more natural NREM sleep to emerge (Uygun et al., 2016). Fitting with these experiments, acute optogenetic silencing of histamine neurons *in vivo* with Archaerhodopsin causes awake mice to transition to NREM sleep (Fujita et al., 2017). Consistent with the experiments on selectively-zolpidem-sensitive neurons producing natural NREM sleep (Uygun et al., 2016), the EEG power of the NREM sleep evoked by opto-silencing of histamine neurons also resembles the power found in natural NREM sleep (Fujita et al., 2017).

## NA and Wakefulness

If selectively inhibiting the histamine system by zolpidem leads to sleep (Uygun et al., 2016), then something similar might happen when the LC, which is the main pathway that provides the neuromodulator NA into the neocortex, is also repressed. The LC comprises a group of noradrenergic neurons with widespread projections (Berridge and Waterhouse, 2003; España and Berridge, 2006; Sara and Bouret, 2012; Carter et al., 2013; Schwarz and Luo, 2015; Aston-Jones and Waterhouse, 2016). Similar to the histamine system, the LC is wake-active, and its neurons start to fire just before the onset of wake (Saper et al., 2010; Takahashi et al., 2010). During active wakefulness the LC neurons fire at less than 6 Hz, less so during quiet wakefulness, and are silent during NREM and REM sleep; just before the onset of NREM sleep, their activity ceases (Takahashi et al., 2010).

An individual LC neuron send projections to many different brain areas (e.g., preoptic hypothalamus, olfactory bulb, cortex and cerebellum), emphasizing that these noradrenergic neurons will globally influence brain state by releasing NA simultaneously throughout the brain (Schwarz et al., 2015). LC neurons fire when an animal is exposed to environmental novelty, and LC activity helps the consolidation of memories in the hippocampus, although surprisingly this effect is due to dopamine release from LC axons, rather than NA (Takeuchi et al., 2016). LC neurons can also co-release other compounds, such as galanin peptide, generally considered as an inhibitory peptide. NA acts on three classes of metabotropic receptor, α1, β and α2 (Bylund et al., 1994; Hein, 2006). The α1 and β receptor class promote excitatory second messenger actions, but the noradrenergic system has an inhibitory component—the α2 type receptors (see “The α2 Adrenergic Sedatives Dexmedetomidine, Guanfacine and Xylazine” section).

There have been many studies on lesioning the LC. The collective result is that long-term LC-lesioned animals have normal wakefulness and sleep (Jones et al., 1977). Straight after an LC lesion, rats have a decrease in waking as measured by the EEG, but over a 4 day period they recover normal levels of wakefulness (Lidbrink, 1974), again illustrating the remarkable plasticity of the brain, and the interconnected and plasticity of brain modulatory systems. Indeed, a triple lesion of basal forebrain cholinergic neurons, histaminergic neurons and LC neurons using saporin-conjugated neurotoxins did not alter the sleep-wake cycle of rats, the only difference being the rats slept more at the light-dark transition of the 12:12 light dark cycle, instead of becoming more active (Blanco-Centurion et al., 2007). There are still some cholinergic, histaminergic and noradrenergic neurons left in these animals, and given their extensive projections, this could account for the lack of effect.

The clearest “sleep phenotype” in long-term LC lesioned animals is that they fall asleep more quickly in a novel environment (Gompf et al., 2010), a result also found when GABA_A_ receptor inhibition is genetically removed from histamine neurons (Zecharia et al., 2012). Knockout of the dopamine β-hydroxylase gene in mice, and hence eliminating NA, also does not alter sleep-wake cycles, again implying compensation and redundancy (Hunsley et al., 2006). In contrast to the chronic lesions, acute manipulation of the LC gives different results (Carter et al., 2010, 2013). Stimulating the noradrenergic LC optogenetically at 5 Hz induces arousal (Carter et al., 2010), and can trigger wakefulness from NREM sleep (Carter et al., 2010) and activating LC neurons chemogenetically with the clozapine-N-oxide (CNO)-Designer Receptors Exclusively Activated by Designer Drugs (DREADD) system can trigger arousal from general anesthesia (Vazey and Aston-Jones, 2014).

As with histamine, NA is a modulator acting across the whole brain: NA has simultaneously excitatory and inhibitory (by the α2 receptors) actions on neurons, astrocytes, microglia and capillaries (O’Donnell et al., 2012). Ironically, although the NA system produces wakefulness and deepens cognition, selectively pharmacologically activating the inhibitory noradrenergic receptors with α2-selective ligands, e.g., dexmedetomidine (DEX), produces deep NREM sleep, hypothermia, and vascular dilation. So, within the arousal-promoting function of NA there is a latent hypothermic and sleep-promoting mechanism too. Most importantly, the state induced by DEX is a type of deep but arousable sedation (Maze et al., 2001; Venn and Grounds, 2001).

## The α2 Adrenergic Sedatives Dexmedetomidine, Guanfacine and Xylazine

For sedation in hospital intensive care clinics, a deeper form of sedation, “cooperative sedation”, is required than the sleep obtained with zolpidem. For this purpose, the α2 adrenergic agonist DEX is popular, and given intravenously (MacDonald et al., 1988; Virtanen et al., 1988; Maze et al., 2001; Venn and Grounds, 2001; Jakob et al., 2012; Adams et al., 2013; Akeju and Brown, 2017; Weerink et al., 2017); in the USA DEX is known by its trade name Precedex, and in Europe as Dexdor (Weerink et al., 2017). DEX produces a NREM sleep-like state in rodents (Seidel et al., 1995; Bol et al., 1997; Gelegen et al., 2014), and a NREM stage 2-like sleep in humans (Huupponen et al., 2008; Mason et al., 2009; Akeju and Brown, 2017). Patients sedated with DEX have reduced risk of developing delirium, a state of mental confusion and exhaustion that often develops with sedatives given chronically in the intensive care unit (Ramsay et al., 1974).

DEX, xylazine and guanfacine, all potent sedatives, are selective agonists at α2 adrenergic receptors (Virtanen, 1989; Savola and Virtanen, 1991; Jasper et al., 1998)—metabotropic G-protein coupled receptors coupled primarily to G_i_ proteins (Bylund et al., 1994; Jasper et al., 1998). There are three receptors α2 receptor subtypes expressed in the brain: α2a, α2b and α2c (Hein, 2006). Being mostly G_i_-coupled, α2 adrenergic receptor activation is generally inhibitory, reducing cAMP synthesis, and causing opening of K^+^ channels or inhibition of AMPA glutamate-gated channels (Aghajanian and VanderMaelen, 1982; Franks, 2008; Ishii et al., 2008; Lur and Higley, 2015). Most often, the α2 receptor class is found on neuronal terminals, including noradrenergic LC axons, but also on many other types of neuron, and acts to reduce transmitter release (Matsuo et al., 2003; Shields et al., 2009; Hara et al., 2016). The prototype drug clonidine also produces sedation and works through these α2 adrenergic receptors, but activates other types of metabotropic receptor too.

Only the α2a receptor is needed for DEX’s and other α2 agonists sedative, hypothermic and antinociceptive actions. Mice with a functional α2a knockout (actually a point mutation in the amino acid sequence that renders the receptor dysfunctional) cannot be sedated with DEX, nor can DEX induce hypothermia or block painful sensations in these animals (Hunter et al., 1997; Lakhlani et al., 1997; Malmberg et al., 2001). In the α2B and α2C knockout mice, DEX still induces full sedation, hypothermia and antinociception (Hunter et al., 1997), ruling these receptors out as targets for DEX’s sedative actions. In fact, even a heterozygote of the α2a knockout mouse is insensitive to DEX (Tan et al., 2002), revealing a pronounced haploid insufficiency of the allele. Half the amount of wild-type α2a mRNA provides insufficient receptor. A downstream ion channel target for DEX’s actions via α2a receptors is the TASK1 potassium leak channel (Linden et al., 2006): DEX’s ability to induce sedation and hypothermia is diminished significantly in mice lacking TASK1 channels (Linden et al., 2006).

## Challenging the Assumption That α2a Autoreceptors on the LC Mediate the Sedative Actions of α2 Adrenergic Agonists

**Figure 3:**
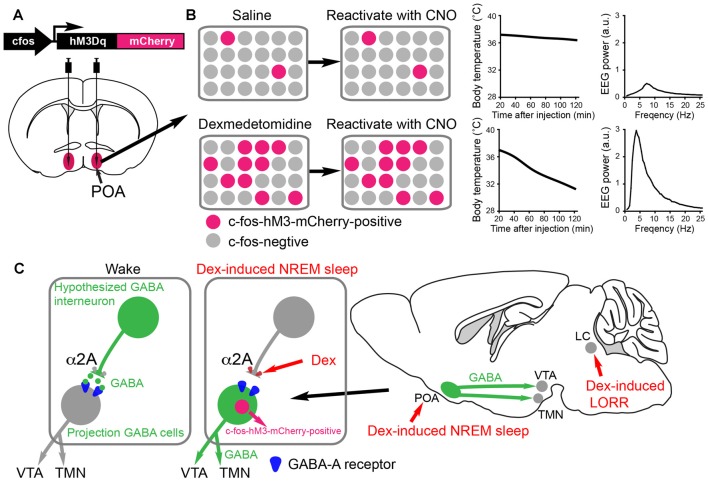
Activity-tagging demonstrates that the α2 adrenergic agonist dexmedetomidine (DEX) induces NREM sleep and hypothermia by activating neurons in the POA of the hypothalamus. **(A)** The activity-tagging system is shown as a simplified construct of a *c-fos* promoter-linked to a DREADD receptor hM3Dq-mCherry reading frame. This DNA construct is transduced into neurons of the preoptic hypothalamus by viral injection. **(B)** A saline injection into these mice produces or does not change the background of c-fos-hM3Dq receptor expression in the preoptic neurons. Thus when a few days after the saline injection the mice are injected with clozapine-N-oxide (CNO), the few excited neurons cause little change in either the vigilance state of the mice or their body temperature (right hand-graphs show temperature and electroencephalogram (EEG) power spectrum). When the mice are given a sedative dose of DEX it excites neurons in the POA, causing these to command a NREM-like state and hypothermia (for example, the NREM sleep could be induced by inhibitory projections to the histamine area, the TMN, or the VTA. In the DEX-activated neurons of the POA, a pulse of *c-fos* driven hM3Dq receptor is made during the DEX induced-sedation. When CNO is given to the mice a few days later, the tagged preoptic neurons are reactivated/excited by CNO and induce NREM sleep and hypothermia. Giving CNO to naïve mice not expressing the CNO receptor has no effect on the EEG or body temperature (Zhang et al., 2015). **(C)** Our hypothesized mechanism for DEX to act in the hypothalamus by dis-inhibition of local sleep-promoting GABAergic neurons. These local GABA neurons are predicted to inhibit the GABA neurons which induce NREM sleep by sending projections to the ascending aminergic neurons. DEX activates α2a receptors on, for example, the terminals of the local inhibitory GABA neurons to reduce GABA release onto GABA projection neurons. The GABA projection neurons would then be more excitable and could inhibit the ascending arousal neurons (e.g., histamine neurons in the TMN area and dopamine neurons n the VTA). The disinhibition of these GABAergic projection neurons causes them to express the *c-fos* gene, allowing them to be activity-tagged.

There is a widespread assumption in the field that α2a autoreceptors on the LC neurons are responsible for DEX’s, clonidine’s, guanfacine’s and xylazine’s actions as sedatives, switching off or dampening down NA release from the LC. Indeed, DEX and clonidine both inhibit the LC neurons through α2 receptors (Aghajanian and VanderMaelen, 1982; Lakhlani et al., 1997; Zhang et al., 2015)—knocking α2a receptors down or out in the LC abolished DEX receptor-mediated inhibition of these neurons (Lakhlani et al., 1997; Zhang et al., 2015). But, NREM-like sedation (increased δ power, immobility, lower body temperature) is still induced by DEX in rats with noradrenergic transmission mostly abolished because of toxin depletion of noradrenergic stores (Segal et al., 1988), and in dopamine-β- hydroxylase-knockout mice or α2a receptor knockout animals (Gilsbach et al., 2009; Hu et al., 2012), or mice with α2a receptors selectively functionally removed from the LC by shRNA knockdown (Zhang et al., 2015). All these findings suggest α2 drugs can work elsewhere in the brain than on the LC to induce the NREM-like sedation. Injection of α2 drugs directly into the POA induces both NREM sleep and hypothermia (Quan et al., 1992; Alam and Mallick, 1994). Therefore, it seems the α2a receptors must be on terminals of neurons other than the LC or on some neurons within the POA. To get at this problem, our lab used *c-fos* dependent activity-tagging (Zhang et al., 2015; Figure 3).

## The Hypothalamic Preoptic Area and α2 Adrenergic Agonist Induced-Sleep

The preoptic hypothalamic area contains a complex mix of wake-active, sleep-active and vigilance-state independent neurons. The area is involved in regulating sleep, wake, electrolyte balance, body temperature and sex amongst other things (Szymusiak et al., 2007; see “More on the Preoptic Area and the Induction of Natural NREM Sleep” section). cFOS expression has been used frequently to define sleep-and wake-active active neurons in the hypothalamic POA (Szymusiak et al., 2007). After, for example, sleep deprivation, the subsequent recovery sleep period has many c-FOS expressing neurons in the POA (Gong et al., 2004). Some of these neurons are believed to be the GABAergic neurons which innervate e.g., the histamine neurons in the TMN area and induce sleep (Sherin et al., 1996; Chung et al., 2017). Similarly, systemic DEX induces excitation in the preoptic hypothalamic area, as seen by cFOS expression (Nelson et al., 2003; Zhang et al., 2015). One study, on the other hand, did not find cFOS was induced in the POA following DEX-induced sedation (Garrity et al., 2015).

A technique known as* c-fos* promoter-based activity-tagging (also known as TetTagging) can be used to ascertain the necessity of neurons for a particular physiological response (Reijmers et al., 2007; Garner et al., 2012). In this method, the *c-fos* promoter is linked to an effector gene, such as the clozapine-N-oxide (CNO)/clozapine-activated DREADD receptor hM3Dq or light-activated channels (Figure 3A). The construct is kept repressed by antibiotic treatments, but when the system is de-repressed by removing antibiotic the *c-fos* promoter can drive effector expression. The effector can then be selectively re-activated (e.g., with CNO/Clozapine on the DREAD receptor) to excite those neurons that were previously *c-fos* active, thus revealing what these physiologically activated neurons regulate. The scheme is shown in a simplified way in Figures 3A,B.

Reactivating neurons that became c-FOS-positive during DEX-induced sedation using the CNO-DREADD system caused the animals to have strong NREM-like sleep (prominent delta oscillations in the EEG) as well as pronounced hypothermia (Zhang et al., 2015; Figure 3B). Thus, the POA neurons were sufficient for DEX’s sedative response (Zhang et al., 2015). These are probably the same neurons that respond to sleep deprivation and command the initiation of recovery sleep, as revealed by serial activity-tagging experiments on the POA (Zhang et al., 2015). We suggest that DEX causes local excitation of these POA sleep-promoting neurons by inhibiting putative upstream GABAergic neurons (see Figure 3C). The identity of these putative interneurons is unknown. Other mechanisms are possible not involving interneurons. In any case, regardless of how they are activated, the neurons disinhibited by DEX could be a new avenue of development of a more precise acting sedative which does not cause hypothermia. In particular, it will be intriguing to see if the DEX-induced hypothermia and sleep can be separated to different subsets of POA neurons.

## α2 Adrenergic Sedative Drugs, Loss-Of-Righting Reflex, and the Brainstem Circuit That Produces REM-Sleep Atonia/Paralysis

As with histamine, NA contributes to the complex phenomenon of wakefulness (Arnsten et al., 2012), but switching off the LC acutely by overstimulation (frequencies greater than 5 Hz), and thus probably depleting transmitter vesicles, does not induce sleep but a frozen state with muscle atonia (Carter et al., 2010). LC neurons receive diverse inputs, but some of the most predominant inputs come from motor areas (Schwarz et al., 2015). In fact, some researchers speculate that although the LC does support the diverse aspects of arousal and wakefulness, these actions are dispensable and replaceable by other modulators, but NA’s most prominent effect is to be permissive for generation of skeletal muscle tone i.e., allowing spinal motor neurons to fire action potentials (Siegel, 2006). Evidence in support of this come from the condition of cataplexy. In cataplexy animals and human patients can suddenly lose postural muscle tone (atonia)—they collapse, but remain conscious and can remember the events. In dogs suffering from a cataplexic episodes, histamine neurons remain active in the cataplexy attack, whereas noradrenergic (and serotonergic) neurons cease discharge or greatly reduce their activity (Wu et al., 1999; John et al., 2004). Thus, the LC neurons are particularly involved in maintaining aspects of arousal concerning postural muscle tone but are dispensable for wakefulness *per se*.

A state termed loss-of-righting reflex, resembling postural muscle atonia, is induced by DEX when the dose of the drug is increased beyond the minimum sedative dose. Loss-of-righting reflex in animals is often considered to be an anesthetic endpoint that correlates with loss of consciousness in humans (Franks, 2008). In the case of DEX, however, we suggest that the specific mechanism for loss-of-righting reflex could be a REM-like atonia/paralysis.

**Figure 4:**
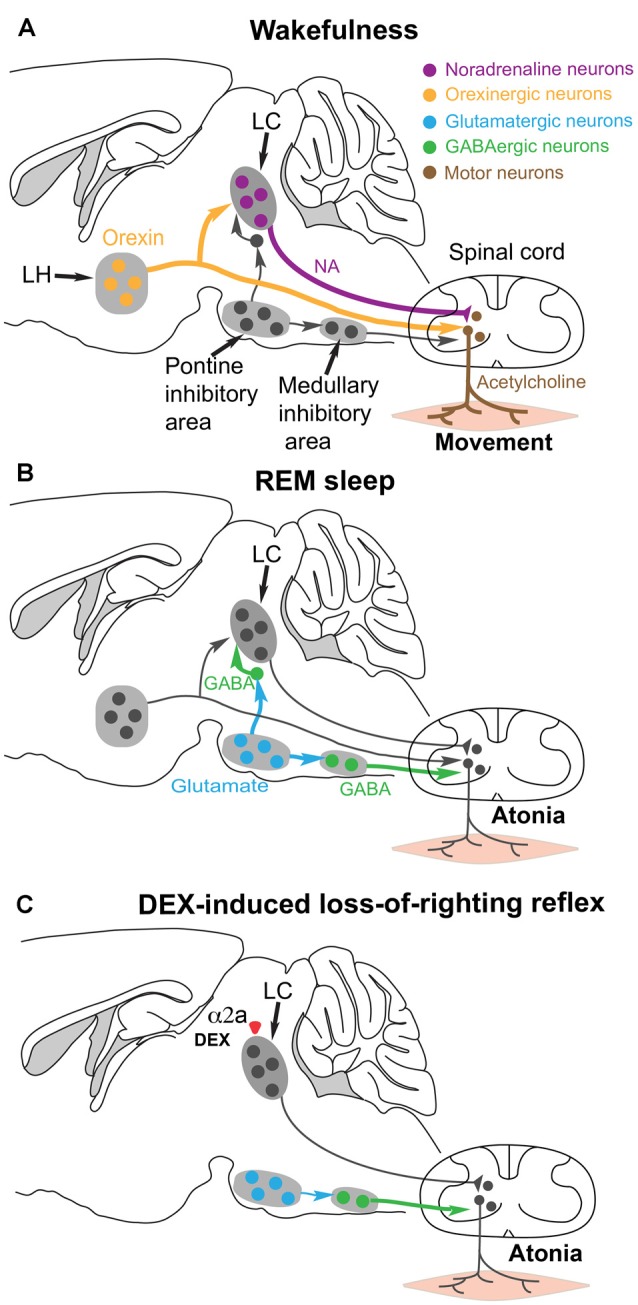
Hypothesis for how the α2 adrenergic agonist DEX could induce postural muscle atonia (and hence loss-of-righting reflex) by engaging the same brainstem circuitry that causes muscle atonia during REM sleep. **(A)** During wakefulness, motor neurons in the spinal cord release acetylcholine onto skeletal muscle to cause excitation and muscle activity. The motor neurons are commanded from the motor neocortex, but receive facilitatory and permissive (dis-inhibitory) neuromodulatory inputs from the noradrenergic locus coeruleus (LC) and orexinergic neurons in the lateral hypothalamus. **(B)** During REM sleep, a group of glutamatergic neurons in the pontine inhibitory area become active and drive GABAergic interneurons in the medullary inhibitory area to silence the noradrenergic neurons in the LC and also the motor neurons in the spinal cord—the net result is muscle atonia. **(C)** We hypothesize that DEX could activate α2a receptors, either on the soma or terminals of the noradrenergic LC neurons to inhibit noradrenaline (NA) release onto spinal motor neurons. This removes the permissive modulatory influence on motor neuron excitation. Not all the circuitry is shown, as it is not known which other cell types have the α2a receptors. Adapted and extended from McGregor and Siegel (2010); Blumberg (2013) and Zhang et al. (2015).

During wakefulness, descending noradrenergic axons from the LC and orexin neurons provide positive modulation of spinal motor neurons that excite the skeletal muscles (McGregor and Siegel, 2010; Rank et al., 2011; Blumberg, 2013; Fraigne et al., 2015; Figure 4A). NA, by activating α1a receptors, promotes the excitability of spinal motor neurons by triggering calcium-mediated-persistent inward currents which, in turn, allow continuous motor neuron firing (Rank et al., 2011). The spinal motor neurons also receive direct commands from descending pyramidal neurons in motor cortex.

During normal REM sleep, the LC neurons are inhibited by GABA neurons that are, in turn, driven by REM-active glutamatergic neurons in the pontine inhibitory region (McGregor and Siegel, 2010; Figure 4B). The REM-active glutamate neurons also drive GABA/glycine interneurons that in turn inhibit the motor neurons. The combined loss of LC and orexinergic excitatory input onto motor neurons, combined with the enhanced GABA drive onto motor neurons during REM produces the atonia (McGregor and Siegel, 2010; Blumberg, 2013; Fraigne et al., 2015). This can also work during DEX induced loss-of-righting reflex: we suggest that if the LC neurons are silenced by DEX activating α2a receptors on the LC, then loss of LC tone could allow inhibition onto the motor neurons to dominate and thus muscle atonia (i.e., paralysis) to appear, manifesting as loss-of-righting reflex (Zhang et al., 2015; Figure 4C).

## Conclusion and Major GAPS in Knowledge

We are far from a consensus about what makes us fall asleep. The homeostatic drive to sleep is expressed as changed neuronal activity (Szymusiak et al., 2007; Zhang et al., 2015), but little is known about how the drive works at the circuit level. In this review, we have seen how sedative drugs, by selectively interfering with two modulatory systems, the histamine system, and the noradrenergic system, produce different types of sleep: fairly natural NREM-like sleep in the case of zolpidem selectively promoting GABAergic drive onto histamine neurons, and a deeper, but arousable NREM-like sleep, by artificially activating the inhibitory arm of the noradrenergic system in the case of adrenergic α2 agonists such as DEX and xylazine. The next step is to dissect the preoptic hypothalamic circuitry to see if the sleep-promoting effects of α2 agonists can be separated from the temperature effects. A sedative that induces deep sedation without the clinical complications of hypothermia could be useful. An important physiological question is how are the preoptic neurons activated by sleep deprivation and the α2 adrenergic sedatives, and is this a common mechanism? Understanding what the sleep drive signal is may reveal the function of sleep and allow the development of the next generation of sedatives.

## Author Contributions

XY, NPF and WW co-wrote the manuscript.

## Conflict of Interest Statement

The authors declare that the research was conducted in the absence of any commercial or financial relationships that could be construed as a potential conflict of interest.
